# Field infection of a gilt and its litter demonstrates vertical transmission and effect on reproductive failure caused by porcine circovirus type 3 (PCV3)

**DOI:** 10.1186/s12917-021-02862-5

**Published:** 2021-04-08

**Authors:** Diana S. Vargas-Bermúdez, Mayra A. Vargas-Pinto, José Darío Mogollón, Jairo Jaime

**Affiliations:** Universidad Nacional de Colombia, Sede Bogotá. Facultad de Medicina Veterinaria y de Zootecnia. Departamento de Salud Animal, Centro de Investigación en Inmunología e Infectología Veterinaria (CI3V)., Carrera 30 No. 45-03, Bogotá, CP 11001 Colombia

**Keywords:** Porcine circovirus 3, PCV3, Putative infection, Reproductive failure, Viral load

## Abstract

**Background:**

PCV3 is a member of the Circovirus family, associated with disease and mortality in pigs. It is not clear whether PCV3 putatively causes clinical symptoms and disease. In the present case, we reported a gilt infected with PCV3 associated with reproductive failures, vertical transmission, tissue lesions, viral replication by in situ hybridization, and the hypothesis that some strains of PCV3 clade one are associated with reproductive failures at the field level.

**Case presentation:**

In May 2019, a pig farm in Colombia reported increased reproductive failures, and the presence of PCV3 in gilts and sows was established in a single form or coinfections, mainly with PCV2 and PPV7. Ten sows with a single infection with PCV3 were found, and one gilt with a pre-farrowing serum viral load above 10^3^ was studied. This gilt was followed up during the pre-farrowing, farrowing period and on her litter for 6 weeks. During dystocic farrowing, a mummy and ten piglets were released, including two weak-born piglets. The highest viral loads for PCV3 were found in the mummy and the placenta. In the weak-born piglets, there were viral loads both in serum and in tissues, mainly in the mesenteric ganglia and lung. Replication of PCV3 in these tissues was demonstrated by in situ hybridizations. PCV3 was also found in the precolostrum sera of piglets and colostrum, showing vertical transmission. The viral load in piglets decreased gradually until week six of life. The viral genome’s complete sequencing was made from the mummy, and its analysis classified it as PCV3 clade one.

**Conclusions:**

This report confirms that PCV3 can cause disease at the field level, and putatively, in this case, we find the generation of reproductive failures. The ability of PCV3 to cause disease as a putative pathogen may be associated with the viral load present in the pig and the strain that is affecting the farm. For this case, we found that viral loads above 10^3^ (4.93 log genomic copies / mL) in the gilt were associated with clinical manifestation and that some PCV3 strains belonging to clade one are more associated with the reproductive presentation.

## Background

Porcine circovirus 3 (PCV3) has been described as a new member of the family *Circoviridae* and the third member of the genus *circovirus* [1]*,* although porcine circovirus 4 (PCV4) has recently been reported [2]. The first reports of PCV3 were made in the USA in 2015 [3] and later, its presence was described as associated with various clinical manifestations in other regions such as Europe [4–6], Asia [7–10] and South America [11, 12]. One of the most relevant aspects of PCV3 is that it has been possible to identify it in many species. Although there is no evidence of clinical signs in them, it does show that the virus has a high degree of adaptation and the ability to transmit between species, which has possibly contributed to its spread, without being able to determine its real impact on the pig industry and even on human health [13–15].

As already mentioned, it has not been possible to elucidate a specific clinical picture since it was only recently that the virus was isolated [16, 17]. The first reports associated the presence of PCV3 with porcine dermatitis and nephropathy syndrome (PDNS) and reproductive failure [3], and later, other reports identified the virus in clinical pictures as diverse as encephalitis [18], pneumonia [19], vasculitis, myocarditis [20] and congenital tremor [21]. Additionally, PCV3 was found in a wide variety of parenchymal tissues [22], blood, colostrum [23], oral fluids [24, 25], as well as fetuses, mummies [25, 26], stillbirths and placenta [27]. The virus can also be found in clinically healthy pigs [28], making the detection of PCV3 not necessarily associated with clinical signs or lesions, and it is widespread for the virus to be detected in the presence of other infectious agents [18, 29, 30]. In Colombia, PCV3 was first reported in 2018 from pig samples with a clinical picture similar to that described for porcine circovirus 2 (PCV2) [12], and then we determined that the virus was spread throughout the country and its presence dated from 2015 (unpublished data).

This report originates from a pig farm located in the central-western region of Colombia in 2019. This farm reported an increase in reproductive failures compared to the previous year. The evaluation of various pathogens was made on a group of 50 pregnant sows between sows and gilts. We only found PCV3 in six sows and four gilts, one gilt being striking where we found a serum viral load of 7.4 × 10^3^ (5.8 log genomic copies (lgc) / mL) at farrowing. We followed up on this gilt, and it was possible to demonstrate by qPCR, histopathological, and in situ hybridization the vertical transformation of PCV3, its participation in reproductive failure, and its classification as PCV3 clade one.

## Case presentation

In May 2019, a commercial sow farm in the central-western region of Colombia reported a reproductive failure associated with mortality in pregnant sows, decreased farrowing rate (85,2% compared to historical of 88%), increased stillbirth rate (4,7% for 2017 and 6,5% in 2018), mummy rate (5,7% for 2017 and 7% for 2018), abortions, and the birth of weak-born piglets. The farm operatd a segregated production system (site one and two in one place, site three in another location), and its vaccination plan for PCV2 consisted of vaccinating female replacements at 20 weeks of age and piglets at day 21 of age. Additionally, the vaccination program included *mycoplasma*, parvovirus, *leptospira*, *erysipelas*, and *Haemophilus*.

To determine the causes, we took serum samples from ten gilts and 40 sows of different parity to assess the presence of pathogens associated with reproductive failure, including PCV2 and PCV3, porcine parvovirus 1 and 7 (PPV1 and PPV7), atypical porcine pestivirus (APPV) and porcine reproductive and respiratory syndrome virus (PRRSV). Ten milliliters of blood without anticoagulant was taken for each female and the samples were transported to the animal virology laboratory of the Facultad de Medicina Veterinaria de la Universidad Nacional de Colombia, Bogotá. Immediately the serum was obtained, DNA and RNA extraction were done with the High Pure Viral Nucleic Acid kit (Roche®) to evaluate by PCR and RT-PCR the presence of the viruses mentioned above. Of the 50 females evaluated, 40 presented different types of infection and coinfections: 12/50 females (24%) PCV2; 5/50 (10%) PCV2 / PPV7 coinfection; 12/50 (24%) PCV2 / PCV3 coinfection and one had a triple PCV2 / PCV3 / PPV7 coinfection (2%). All were negative for PRRSV. Four gilts and six sows (10/50; 20%) were positive only for PCV3. Of this group, nine females presented viral titers below 10^2^, and their viral activity we not permanently monitored. However, they are part of a complementary study of coinfections in the farm under investigation (unpublished data). We chose and followed a gilt that presented for PCV3 a viral titer in the pre-farrowing serum of 7,4 × 10^3^ (5,8 lgc / mL) and at farrowing presented dystocia, a mummy, a litter of ten piglets including two weak-born piglets that died a few hours later. Additionally, one piglet died at week three, and another was euthanized at week five, both with waste symptoms. The presence of PCV3 was determined from samples that were classified as follows: (i) samples from the gilt: pre-farrowing serum, post farrowing serum, nasal swab, vaginal swab, colostrum, placenta and mummy; (ii) samples of the weak-born piglets: spleen, mesenteric lymph nodes, tonsils, liver, kidney, heart and lung; (iii) litter samples: sera from birth to week six, including precolostrum sera. For this purpose, we extracted the DNA with the High Pure Viral Nucleic Acid kit (Roche®) and did a conventional PCR using primers F (5′-GGGCACACAGCCATAGAT-3’) and R (5’-TTCCGGGACATAAATGCT-3′) reported by Chen et al. 2017 [21], obtaining a product of 267 bp. The thermal protocol used started at 95 °C for 5 min, followed by 35 cycles 95 °C for 30 s, 58 °C for 40 s, 72 °C for 40 s and 72 °C for 5 min. We found PCV3 in the gilt in pre and post-farrowing serum, nasal and vaginal swabs, placenta, and colostrum; it was also found in mummy tissues and pre and post-colostrum sera from weak-born piglets and piglets. Subsequently, to quantify the viral load of PCV3, we applied a qPCR using the Light Cycler® 480 II Roche kit. Briefly, a 267 bp fragment was amplified from PCV3 strain COL/Cundinamarca2/2018 (Genbank: MH327785), and the amplicon was cloned into the TOPO-TA cloning (ThermoFisher Scientific®) according to the manufacturer’s instructions. The PCV3 recombinant plasmid was sequenced at the commercial sequencing facility SSiGMol (Servicio de Secuenciación y Análisis Molecular, Instituto de Genética, Universidad Nacional de Colombia). We performed qPCR using the same primers as for conventional PCR. The reactions were performed in 20 μL volume containing 50 ng DNA, 10 μL of 2X SsoAdvanced Universal SYBR Green Supermix (Bio-RAD®) and 0.4 μM of each forward and reverse primers. The assay was performed using a Light Cycler® 480 II-Roche thermal cycling system under the following cycling conditions: 95 °C for 5 min followed by 40 cycles of 95 °C for 1 min, 60 °C for 25 s and 72 °C for 5 s. Melting curves were generated by monitoring SYBR green signal’s fluorescence from 70 °C to 95 °C. Negative control contained ddH2O, and the positive control was the PCV3 plasmid. All reactions were conducted in triplicates. The 10-fold serial dilutions evaluated the limit of detecting the SYBR green-based real-time qPCR from 1,15 × 10^1^ to 1,15 × 10^9^ copies / μL. The quantification cycle (Cq) values range from 8,98 to 35 cycles with a linear correlation (R2) of 0,989 (slope = − 3514) between the Cq value and the logarithm of the PCV3 copy numbers.

**Fig. 1:**
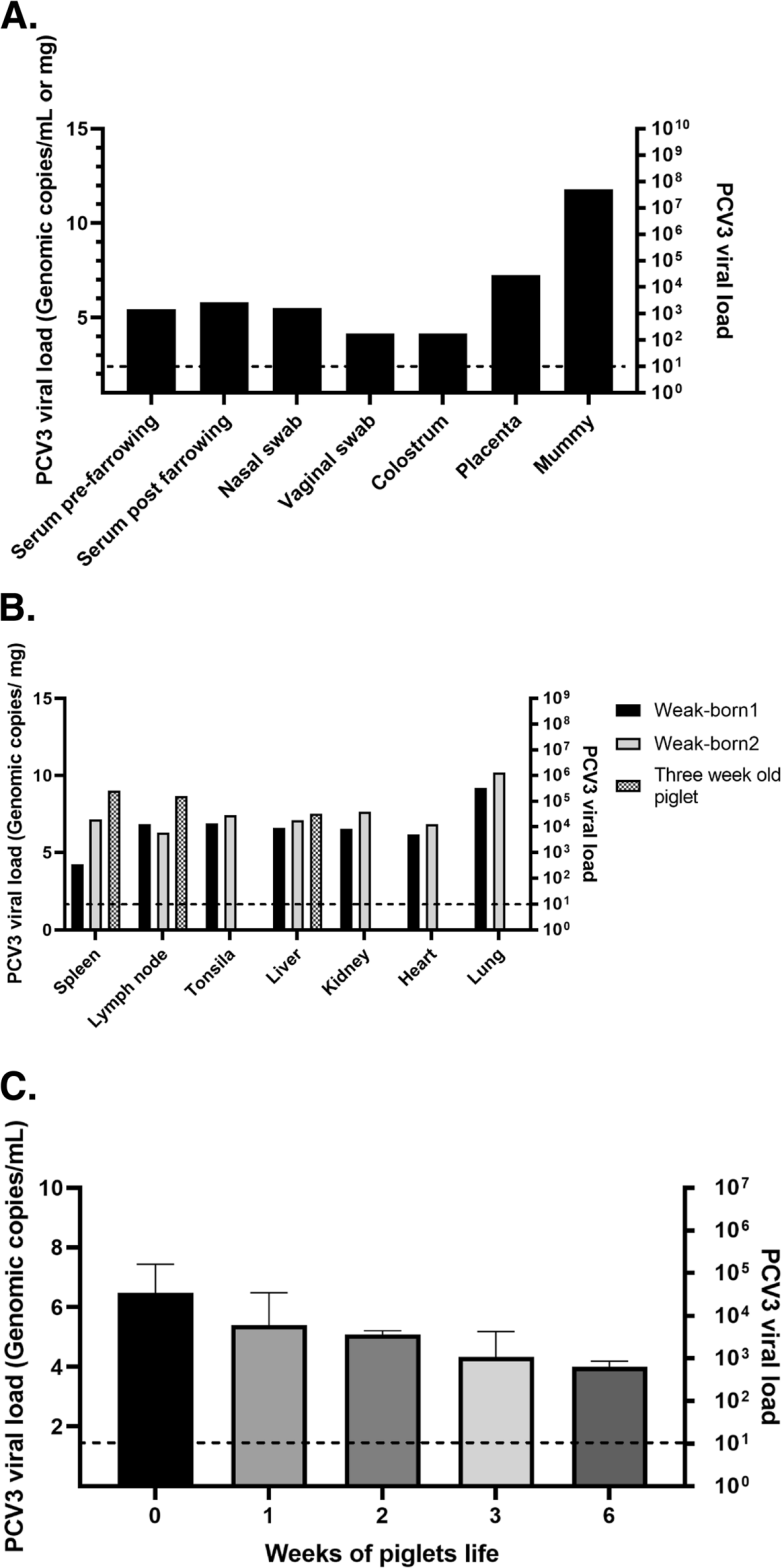
Viral loads present in the gilt and its piglets. **a** Viral load in samples from the gilt. **b** Viral load in tissues obtained from two weak-born piglets and one dead piglet at week three. **c** Viral load in the serum of the piglets from the day of farrowing until week six

**Fig. 2:**
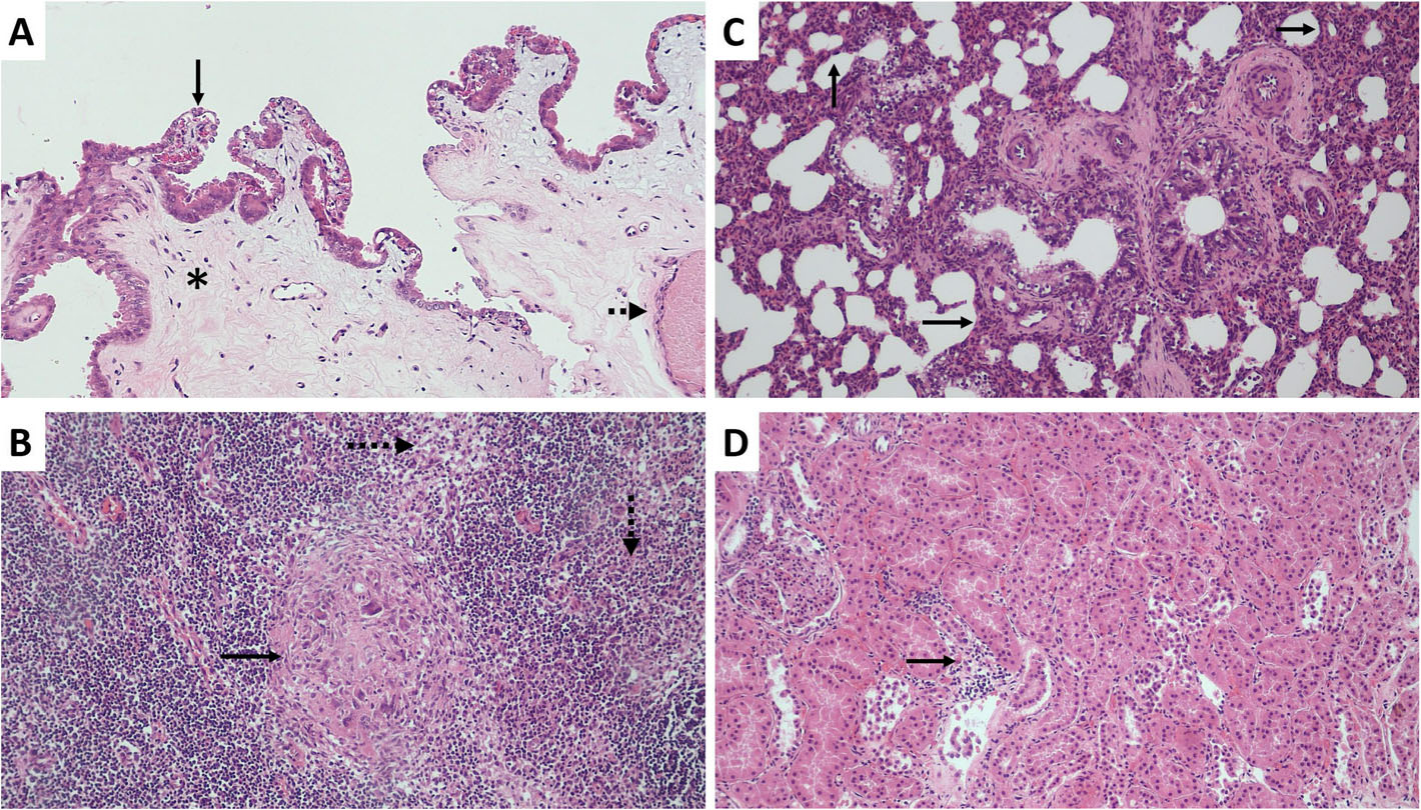
Histopathology of tissues from the gilt and weak-born piglets, processed with hematoxylin and eosin staining. 20x magnification. **a** Placenta with necrotic changes in the epithelium (continuous arrow), severe congestion (dotted arrow), and incipient submucosa-lymphoplasmacytic inflammatory infiltrate (asterisk). **b** Mesenteric lymph node with moderate lymphoid depletion (dotted arrow), presence of a histiocytic infiltrate and some giant cells, Formation of a small microgranuloma (continuous arrow). **c** Lung with moderate multifocal thickening of the alveolar septa associated with congestion and lymphoplasmacytic mononuclear inflammatory infiltrate. Depletion of lymphoid tissue associated with the bronchi. **d** Kidney with moderate inflammatory lymphoplasmacytic infiltrate (interstitial nephritis), with slight focal desquamation of the tubular epithelium

**Fig. 3:**
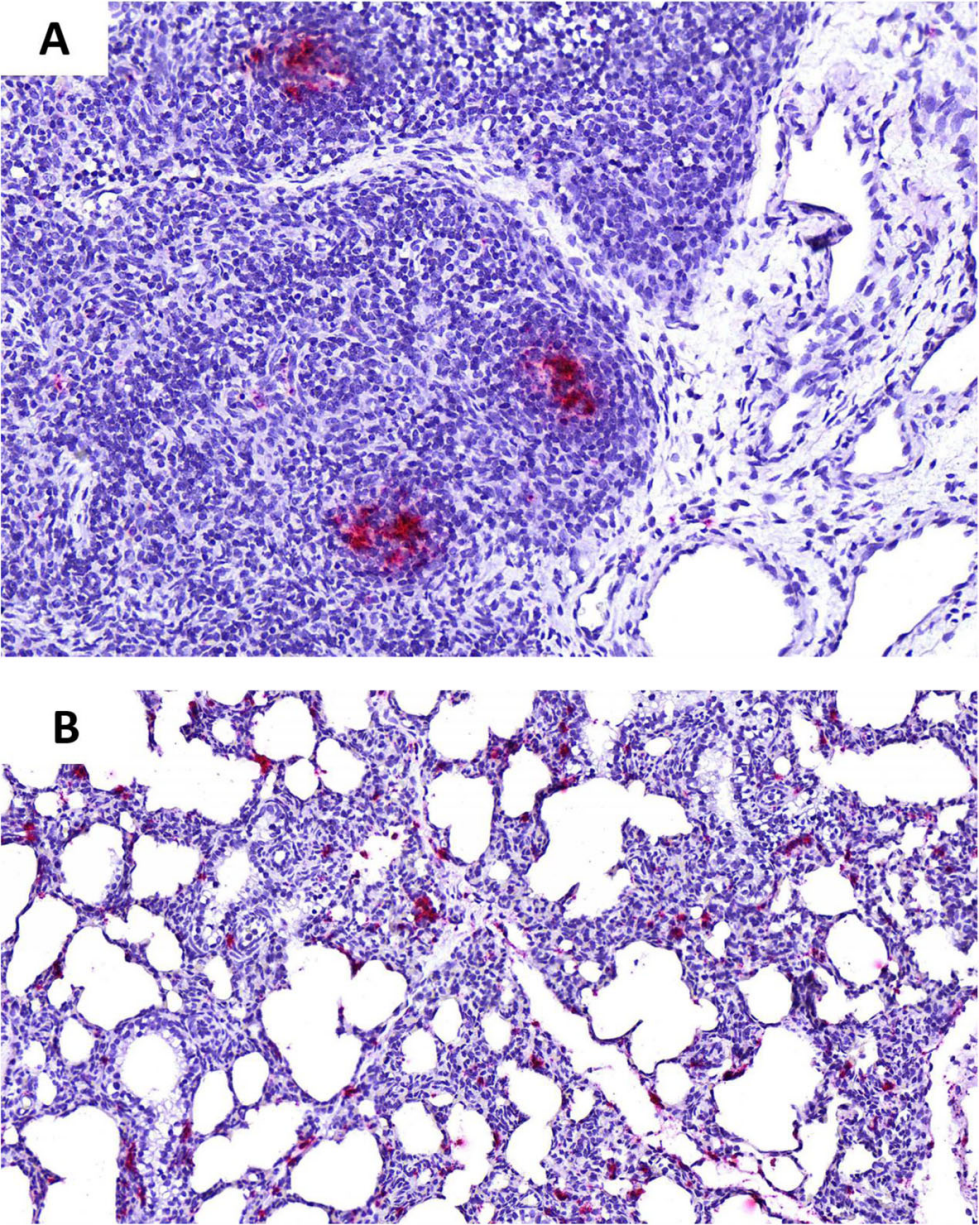
RNAscope in situ hybridization (ISH) on tissues from a weak-born piglet. **a** PCV3 replicating was demonstrated in lymphoid follicles of a lymph node that had depletion. Hematoxylin counterstain (20x). **b** PCV3 mRNA (red) was detected in the cytoplasm of cells infiltrating the lung septal interstitium (may be macrophages). Hematoxylin counterstain (20x)

Viral loads of PCV3 were detected in all the samples taken on the day of farrowing, the highest being those found in the placenta and in the mummy with titers of 2 × 10^5^ y 7,5 × 10^9^ (7,23 and 11,8 lgc / mL) respectively (Fig. 1a). As for the two weak-born piglets, who died post-farrowing from the moment of birth, they manifested severe respiratory distress and inability to suckle; in them, viral loads of PCV3 in serum 5,42 × 10^6^ and 1 × 10^7^ (8,64 and 8,93 lgc / mL) were found. Regarding the tissues evaluated in these piglets, we never found viral titers below 10^3^ (4,93 lgc / mL), with remarkably high viral load in mesenteric lymph nodes (5,19 × 10^4^) (6,64 lgc / mL) and lung (7,7 × 10^7^) (9,81 lgc / mL), this last value coinciding with the clinical picture, histopathological findings and ISH (Fig. 1b). Additionally, the viral load was determined over time in the piglets of the litter, showing a progressive decrease from farrowing, where the precolostrum sera values were between 10^3^ y 10^6^ (4,93 and 7,93 lgc / mL), and from the third week, they decreased, finding values below 10^3^ (Fig. 1c). In parallel, tissue samples were fixed in 10% buffered formalin. These were processed with the routine technique for light microscopy and were stained with hematoxylin and eosin. In most of the tissues evaluated, we found significant changes. The placenta showed necrotic changes, congestion, and inflammatory infiltrates (Fig. 2a). In the tissues from the two weak-born piglets, lymphoid depletion and granuloma formation were found in the mesenteric lymph node (Fig. 2b), in the lung, a moderate multifocal thickening of alveolar septa (Fig. 2c) and the kidney interstitial nephritis (Fig. 2d). To corroborate the presence of PCV3, ISH was applied. For this purpose, we sent the tissue blocks to the Veterinary Diagnostic Laboratory, University of Minnesota. RNA ISH was performed using the RNAscope® 2.5 HD Reagent Kit -RED and the RNAscope® 2.5 HD Reagent Kit - BROWN according to the manufacturer’s instructions for formalin-fixed paraffin-embedded samples. ISH detected PCV3 in a weak-born piglet, and the most abundant labeling (PCV3 replicating) was found in sections of the lung and lymphoid follicles of a lymph node that depleted (Fig. 3a and b).

**Fig. 4:**
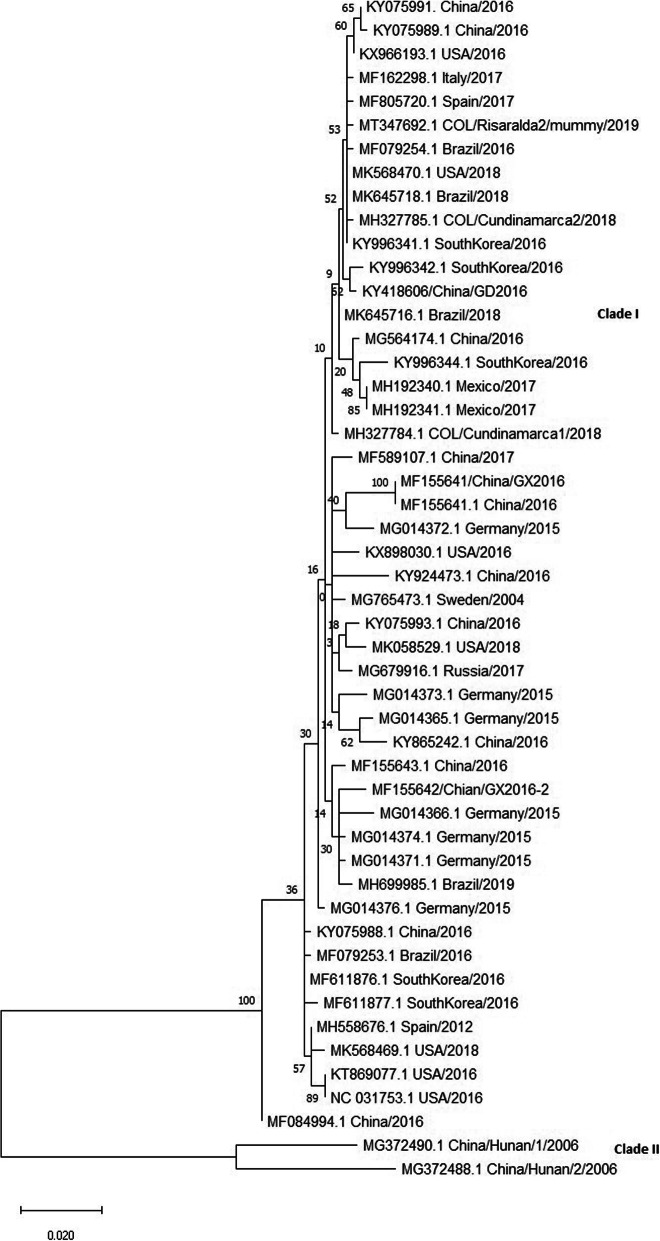
Phylogenetic analysis based on full-length Cap (ORF2) nucleotide sequences of a PCV3 isolate from a mummy and 48 reference strains. There are also two sequences reported in the tree from Colombia (MH327784.1 and MH327785.1), including the one reported in this study (MT34769.1 COL / Risaralda / mummy / 2019). Maximum Likelihood tree based on the nucleotide of the Cap reconstructed using MEGA 7 software. The clade of trees was estimated with 1000 replicates of bootstrap (BT) analysis. All PCV3 strains were divided into two clades: PCV3 clade one and PCV3 clade two based on the classification of Franzo et al. 2020 [31]

The PCV3 full genome from the mummy sample was obtained using four sets of specific sequencing primers reported by Palinski et al. 2017 [3] and following the thermal profile described by Vargas-Bermudez et al. 2019 [12]. PCR products were directly sequenced in both directions at the commercial sequencing facility SSiGMol (Servicio de Secuenciación y Análisis Molecular, Instituto de Genética, Universidad Nacional de Colombia). The DNA sequence was aligned against 48 sequences reported in the Genbank and classified according to what was proposed by Franzo et al. 2020 [31]. All Phylogenetic analyses were performed by using MEGA 7 [32]. Maximum likelihood trees were constructed using the p-distance model and were evaluated with 1000 bootstrap replicates to analyze the clustering’s robustness (MEGA7). The complete genome sequence of PCV3 has been deposited in GenBank under the accession number MT347692. All sequence was translated, and PCV3 clustering was evaluated based on the amino acid composition of the ORF2 (Fig. 4), and it was shown that the strain found in the mummy was classified in the PCV3 clade one.

## Discussion and conclusions

The participation of PCV3 as a putative cause of the disease is a topic of a broad discussion, and its verification difficult by the limitation of reproducing the disease under controlled conditions since the virus is difficult to isolate. In the present report, we studied a farm with reproductive failures with PCV3 and coinfections, and within this, a gilt that presented infection with PCV3 was chosen as the sole cause of clinical symptoms, and we wanted to determine the behavior of the virus at the field level. The presence of PCV3 coinfections with other viruses is expected at the farm level as it is also possible to find infection with the unique company of PCV3. In this sense, farms can have variable ranges of PCV3 positivity within them; in our case, by PCR, we found that of 50 evaluated females, ten presented unique infection to PCV3 (20%). Reports in other geographical areas show that it is possible to find within a farm the presence of viral co-infections and unique infections with PCV3, which has been demonstrated by ELISA [33], PCR [34–36], qPCR [33, 37], and metagenomic [18]. The most notable clinical manifestation in the studied farm was reproductive failures represented by decreased farrowing rate, increased stillbirth rate and mummy rate. There were no reports of symptoms such as porcine dermatitis and nephropathy syndrome (PDNS) or respiratory disorders described for PCV3 by many authors. In the sense of reproductive clinical manifestations by PCV3, our results may be in agreement with Zou et al. 2018 [38], Who found that the PCV3-positive rate was significantly higher in sows with reproductive failure (45.9%) than in healthy sows (21.9%). There are very few studies where the clinical effect of PCV3 has been determined as a putative cause of the disease, the only two reports where this was determined were in the USA. Arruda et al. 2019 [18] determined that PCV3 mainly caused the reproductive failure, which coincides with our report, although they also found PDNS and periarteritis. Additionally, Mora-Díaz et al. 2020 [17] managed to isolate the virus from reproductive cases such as weak-born pigs or elevated stillborn and mummified fetuses, a symptom that coincides with that found in our study. They also report isolations and injuries from multiple tissues, including lung, kidney as in our case, and the heart and brain. Although the origin of the isolates was from reproductive failure, the virus’s effect was not analyzed. On the other hand, in China, Jiangen et al. 2019 [39], experimentally inoculated piglets with a PCV3 DNA clone to reproduce the disease, achieving the presentation of respiratory symptoms and dermatitis. Although all these studies indicate that PCV3 induces clinical symptoms, it is also essential to keep in mind that its severity and variability depend on PCV3-positive rates proposed by Xu et al. 2018 [40].

No field-level studies are found in the literature that tracks a PCV3-infected sow as a putative cause of disease and determines the effect during the perinatal period. In our report, the virus’s presence in pre and post farrowing serum, nasal and vaginal swabs, colostrum, and placenta were found in the gilt studied on the day of farrowing. The highest viral load found was in the placenta (2 × 10^5^) (7,23 lgc / mL), indicating a high tropism of PCV3 for this tissue, which would explain the effect on reproductive failure by the presentation of mummies, weak-born piglets, and infected piglets. Apparently, for PCV3, the effect on reproductive failure and its severity is closely linked to the sow’s viral load during pregnancy and could be an element to take into account to control the clinical effect of the virus. There are also no studies showing the effect of a high PCV3 viral load on the placenta, particularly towards the end of gestation, and there are few that demonstrate the presence of the virus in this tissue. Here it is essential to indicate that this gilt also presented dystocia that could be attributed to the effect of PCV3 on the placenta. There are not many studies on this aspect, and even Hayashi et al. 2018 [9] evaluated PCV3 in multiple pig tissues in nine prefectures in Japan and found no presence of PCV3 in placentas. In the present study, we also proved that PCV3 is transmitted vertically and is directly involved in reproductive failure, which had been suggested by [3, 41]. This was proven since the live-born piglets showed the highest viral titers on the day of farrowing (precolostrum serum), and these gradually decreased over 6 weeks. Likewise, the effect of vertically transmitted PCV3 was manifested in the weak-born piglets that died hours later and in the two piglets that developed marked deterioration and were slaughtered at weeks three and five of life. Reproductive failures, as previously mentioned, have been reported by various authors; what was not determined is the dynamics of PCV3 in piglets by vertical transmission. Interestingly, our study shows that the viral load in piglets decreased over time. This was indeed caused by maternal antibodies, similar to that demonstrated for PCV2 [41]. What is striking here is that viral load by vertical transmission could be a determining factor for a clinical effect. In our case, with a viral load above 105 (6,93 lgc / mL) in some piglets, the clinical manifestation (loss and death) could not be controlled by passive immunity. On the gilt side, it can be argued that serum viral loads on the day of farrowing above 103 (4,93 lgc / mL) could indicate a clinical effect in the litter, such as weak-borns and progressive weight loss. The presence of clinical symptoms associated with viral load in PCV3 has already been proposed by [38, 40] and, conversely, Klaumman et al. 2018 [42] propose that no significant correlation was found between the detection of PCV3 and any production phase nor clinical/pathological condition. Another aspect to consider is the presence of viral titers of 10^2^ (3,93 lgc / mL) in the gilt’s colostrum. If piglets had an average viral load above 10^4^ at birth and received more viruses through colostrum, this could aggravate the clinical presentation, and the neutralizing effect of maternal antibodies could be partial and insufficient to control the disease. In this regard, the transmission of PCV3 via colostrum is well documented by Kedkovid et al. 2018 [23] and coincides with our study as a route of viral dissemination, but it differs in that the colostrum titers for its report were 4.01 to 7,33 lgc / mL while in our report it was 3,93 lgc / mL. Likewise, in the same study [23], it was found that the prevalence of PCV3 shedding in the colostrum of the primiparous sows was 30% higher compared to multiparous sows. Lower parity sows tend to have more positive animals than higher parity sows based on the presence of PCV3 in the serum and colostrum as proposed [28] and as demonstrated for PCV2 [41]. The progressive decrease in viral loads in piglets may indicate the neutralizing capacity, although incomplete in this case, of maternal antibodies. The only report of antibody generation by seroconversion through an experimental infection with PCV3 was made by Jiang et al. 2019 [39] but it is not known what their neutralizing capacity is, and even less in passive immunity.

Histopathological findings found in litter mortality revealed a pneumonic pattern and reticulohistiocytic hyperplasia in the lymphoid tissue. The reports by Deim et al. 2019 [27] and Kim et al. 2018 [19], also rule out the possibility of participation of other pathogens in the described lesions, reinforcing the action of PCV3 as a putative agent. The presence of PCV3 was corroborated by RNAscope ISH, proving strong evidence of productive replication as has been determined in other studies [17, 20, 43]. These results are correlated with the tissues where a higher viral load was found, particularly in the lung and the mesenteric lymph node of the weak-born piglet. The above coincides with that reported by [19], who describe a significant viral load in the lung and lymphoid tissue of piglets infected with PCV3. The determination of active replication in the progeny of the infected gilt corroborates the vertical transmission of the virus.

The phylogenetic classification of PCV3 has evolved since its discovery, and various approaches have been proposed. There is currently a consensus on the classification in two clades (one and two) proposed by Franzo et al. 2020 [31] based on the nucleotide sequence of the ORF2 or amino acids of the cap protein. Based on this, to date, all isolates reported in Colombia, including the one presented in this study, correspond to strains located in clade one. Fu et al. 2018 [44] proposed the old classification based on mutations at amino acids 24 and 27 within the cap protein and proposed three clades: PCV3a, PCV3b, PCV3c. Under this classification, it was possible to propose an association of the PCV3b and PCV3c strains with reproductive cases in the United States [17], while PCV3a did not. With the currently accepted classification [31], these strains are integrated into clade one. It would be essential to determine if, within this clade, isolates could be found associated in a more significant proportion with reproductive failures, as is the case with those obtained in various areas of Colombia (unpublished data).

The results found in the present case and their comparison confirm the presence of PCV3 in Colombia and confirm its possible association with its clinical picture, affirming that PCV3 is a pathogen that affects reproductive failure in gilts. The evidence of vertical transmission, the presence in the litter, and the permanence of the virus in parenchymal tissues can contribute to clarifying the pathophysiological events that facilitate the presence and permanence of the virus on farms. It is advisable to monitor piglets with putative PCV3 infection in the field throughout the production cycle to determine the behavior and effects of the virus after week six of life.

## Data Availability

All data generated or analyzed during this study are included in this published article.

## Abbreviations

APPV: Atypical porcine pestivirus
DNA: Deoxyribonucleic acid
ELISA: Enzyme-linked immunoSorbent assay
ISH: In situ hybridization
PCR: Polymerase chain reaction
PCV2: Porcine circovirus type 2
PCV3: Porcine circovirus type 3
PDNS: Porcine dermatitis and nephropathy syndrome
PPV1: Porcine parvovirus 1
PRRSV: Porcine reproductive and respiratory syndrome virus
qPCR: Quantitative polymerase chain reaction
RNA: Ribonucleic acid
